# Etiology of Convulsions in Children

**Published:** 1883-08

**Authors:** Adolph Kjellberg

**Affiliations:** Sweden


### Original Translations.

ARTICLE V.

ETIOLOGY OF CONVULSIONS IN CHILDREN. By PROF. ADOLPH KJELLBERG. Sweden: Translated by C. W. Johnson, M.D., Chicago.

The so called functional nervous diseases occur very frequently during childhood, and convulsive diseases are especially abundantly represented.

Rythmically, we separate convulsions into two general groups, the clonic and the tonic. Clonic convulsions are those where muscular contraction and relaxation follow each other closely, each action taking up about the same amount of time.

Tonic convulsions are those where the contractions follow each other so closely that the muscles have no time to relax between each contraction, but seem constantly rigid.

To the first group belong those convulsions that come on suddenly, usually in many muscles of the body, followed by a more or less complete unconsciousness in the patient. They attack, by preference, children, most frequently occur unexpectedly, with alarming symptoms; are hence of more than usual interest to the physician, and an explanation of their many different causes is of special importance to him. This is at times very difficult, and at times not at all discovered.

Here we frequently miss our guide that otherwise in complicated diseases solves the problem. I mean pathological anatomy. In how many cases of eclampsia and epilepsy that result in death does not anatomical pathology stand nonplussed and unable to explain the manner of the disease, in defiance of the most thorough investigation? Not at all strange, because within the pathological science of the nerves, many processes take place that cannot be followed with knife or microscope.

Nevertheless, it is another branch of our science that has of late aided the clinical observer to explain obscure questions within the pathology of the nerves; it is the now persecuted experimental physiology. It is this branch we have to thank largely for our knowledge of nervous diseases, and to this branch we are chiefly indebted for what we know about the subject under consideration.

Brown-Sequard, Sciff, Kussmaul and Tenner have thus shown us that the point where convulsions originate is limited to the pons and medulla oblongata; Nothnagel has even located the "convulsive center" in the pons only.

Landois has found that venous hyperæmia in the brain lessens the heart-beats, and when the heart has become very slow general convulsions will result, just the same as in anæmia of the brain.

Hermann and Escher could, by completely stopping the venous blood flowing from the brain, produce general convulsions. Nasse has shown that through an increased amount of carbonic acid gas, and at the same time lessening the amount of oxygen, convulsions may be produced.

Physiology tells us that an increased temperature enhances the excitability of the nerve elements; likewise certain medicines, as strychnia.

Soltmann has shown that the motor centers discovered by Hitzig, are probably not to be found in children, but develop later; furthermore, the excito-motor nerves are wanting for the the large brain, expecially the cortex as the organ for judgment, for the will is in the new born child not yet capable of performing its function; the will has no power over motion; all motor expressions are without knowledge or consciousness, dependent on reflex actions. He has also shown by experiments that the motor nervous irritability in the new born is very small. He has also shown by direct experiments that the irritability of the vagus, as an inhibitory nerve, is less developed in the child than adult.

This is all of especial interest and very important to know. Those experiments indicate to us where the irritation must take place to induce a convulsive attack. We are instructed that convulsions can be induced by anæmia of the brain as well as by hyperæmia. We are taught that an excess of carbonic acid gas in the blood will produce the same result.

It explains to us the long known fact that in children there is a certain disposition to convulsions. This disposition is properly not dependent on any increased irritability in the sensitive or motor system, nor in the reflex center; but it is dependent on the fact that the brain of the child is more or less involuntary, and that it consequently cannot as yet through the will effect or subdue the reflex centers. But when experience further tells us that the new-born infant is not as disposed to convulsions as the child of a few months of age, and that this fact consequently is in conflict with what was just said regarding the so-called increased reflex irritability, then comes again experimental physiology to our aid, and explains to us that the irritability of the motor nerves in the new-born infant must be very small or insignificant.

What are the causes of convulsions in general, i. e. how do they come about? Now we must admit that they come about by an irritation of the place wherein convulsions originate. Experimental physiology has shown us this place to be the medulla oblongata and the pons, or, according to Nothnagel, the latter only. An irritation here must take place; but to bring about a convulsion, must the irritation be abnormally exalted, or the irritability in the so-called convulsion center increased, or both? These conditions may occur simultaneously. The irritability may be direct, i. e., affect the convulsive center directly, or it may be an indirect or reflex irritability. Therefore, convulsions are divided into direct and indirect, central and peripheral, symptomatic and sympathetic, etc. Another category includes the so-called idiopathic convulsions; they come seemingly without motive and we are unable to discover any cause. But causes, of course, must exist, although we cannot discover them, and the more our science grows the fewer must idiopathic convulsions be, until they are things of the past. I, therefore, do not take them up here.

My point of view being clinical, I will divide convulsions into symptomatic and sympathetic. The former corresponds to the so-called direct or central, the latter to the so called indirect or peripheral.

Symptomatic convulsions are those that take place at the beginning of a disease, or during its course, by direct irritation of the convulsive center. This generally happens as a consequence of pathological changes within the blood-vessels, hence this form of convulsions is sometimes called hæmatogenic.

Sympathetic convulsions are those that take place by some irritation of the sensitive nerves; this is conveyed to the convulsive center and a convulsion is produced.

This division of convulsions is not at all satisfactory, for, taken as a whole, several causes coöperate, as we will see hereafter.

Symptomatic convulsions are produced partially by a *disordered circulation*, and partially by a *change in the blood itself*.

Disordered circulation is the most common cause, by producing a sudden anæmia of the brain. If, for example, the circulation of the blood to the brain is suddenly arrested (i. e., in both carotid and vertebral arteries), the phenomena will be as follows: The pupils at first contract, then dilate; the jaws are closed, the eye-balls rotate upward; breathing is difficult and short, later, slow and deep; then violent general convulsions take place with loss of consciousness. If circulation is now again permitted, the subject will be restored to normal state. Such an experiment has been performed by Kussmaul and Tenner. It shows us the whole group of symptoms, and it also shows us the cause to be anæmia of the brain. This anæmia must be suddenly produced, however, to produce a convulsion. Kussmaul and Tenner have shown three things as necessary—viz., loss of blood (to brain) must be large, must occur at one time and suddenly, and the subject must be in good condition.

An anæmia of the brain that has been developed by degrees will not produce convulsions.

An anæmia of the brain to have the power of producing convulsions may be caused as follows:

(*a*) *A sudden loss of blood*, something we often see happen during childhood.

(*b*) *Sudden and severe loss of juices*, as profuse vomiting and diarrhœa.

(*c*) *Arterial contractility*. This certainly plays an important role, by causing anæmia of the brain. For example, psychical impressions, especially those extreme in effect, such as fright, wrath, etc.

It acts probably by its effect and influence on the vaso motor nerves, and causes arterial contractility, anæmia and, lastly, convulsions. Sauvages mentions a case where a child, while in great anger (because it did not get the food it liked), went into convulsions. Swicten reports a case where a child became frightened at a dog; afterwards the child would go into convulsions by seeing a dog, or even hearing one bark. Its is also possible other causes may produce convulsions, such as fright, and this again may produce overaction of the heart, with increased flux of blood to the brain.

(*d*) *Compression of the skull* in infants, pressure on an extended fontanelle, pressure on the back of head in cranio-tabes may all produce anæmia of the brain (cortex), and thus convulsions occur.

(*e*) *Anatomical changes in the brain*, as extravasation of blood, softening, growths, etc., sometimes cause convulsions; just how it is not easy at all times to say. It may be caused by direct irritation of the convulsive center, as in hæmorrhage or growths, but the probability is that by an increased pressure anæmia takes place.

(*f*) During the course of disease, not infrequently there is anemia of the brain, as in hydrocephalus, acute brain diseases, inflammations, fevers, etc.

Just as anæmia of the brain may cause convulsions, so may hyperæmia of the brain be the cause, if not direct, nevertheless indirect. An explanation will probably be in its place, as it may seem curious that two conditions, so different from each other, may produce the same phenomena. Let us consider the circulation, especially that of the lymph within the brain. Through late researches by Key and Retzius, so essentially valuable, we have received an accurate knowledge of the lymphatic vessels in the brain and its membranes. We now know that there is an uninterrupted connection between the sub-arachnoidal spaces of the brain and spinal column, the epi-cerebral spaces and ventricles, and also between the peri-vascular spaces within the brain. Key and Retzius have shown us, that the lymph vessels follow the blood-vessels in the large hemispheres, as well as in the large ganglia (optic thalamus, corpora striata, etc.). The skull forms a closed and firm cavity that will not expand; the contents consist of the brain, blood and lymph vessels. When the circulation is normal, the same amount passes continually to the brain.

In the infant where the sutures and fontanells are yet open, and where the skull may be somewhat expanded as well as reduced, the arterial circulation can be increased somewhat, without increased pressure; so may the venous circulation exceed the arterial without diminishing the pressure within the brain. As the heart's systole and pulse-waves advance must an expansion of the arteries occur, and for the time being an increased amount of blood enters; likewise during expiration the venous blood is hindered and a slight venous stasis results. When the skull forms a firm space and the brain cannot be essentially compressed, then a regulator for the circulation must be found; this regulator is the cerebro-spinal fluid. When the blood current rises, or the venous is hindered, then the cerebro-spinal fluid yields and gives room to the blood. This can only take place within a certain degree; if the arterial current is considerable, then the fluid in the perivascular spaces cannot yield sufficiently. What happens then? Counterpressure, and the consequence is that the capillaries are compressed.

The greater the hyperæmia, the greater the counterpressure; the blood wave cannot be forced to its terminus; consequently, there is a lack of blood in the capillaries—an anæmia in the cortex of the brain, and large ganglia is the result.

During venous stasis, the fluid around the veinous and perivascular spaces is driven back, the pressure on and about the perivascular spaces increases, the arterial wave diminishes, the blood is not driven forward, and thus it happens that the arterial blood supply is diminished ; and thus it is clear that there is an anæmia of the capillaries of the brain. This gives us an explanaation of experiments conducted by Landois showing that a venous stasis may produce a convulsion. We see, also, that hyperæmia and a venous stasis may produce the same result, viz., anæmia of the brain and then convulsions as a result.

A flux to the brain occurs in children very often, more so than in adults. The flux is dependent on the number of heart contractions. We all know how easily and suddenly the function of the heart is aroused; an emotion, a fever, and the heart is at once overactive, usually the younger the child the greater the action. This fact is in harmony with Soltman's view that the younger the child the less developed is that system of nerves (vagus) whose function is inhibitory.

We know how easy a sudden fluxion to the brain may be produced in children, as in many acute diseases, especially fevers, and we have seen that this hyperæmia can produce anæmia of the cortex of the brain ; then we can easily understand why convulsions in similar cases do occur, and also how they are produced.

The ease with which hyperæmia in the infant is produced would probably more often bring disturbing brain symptoms, were it not compensated for by the possibility of the skull expand ing through the open sutures and fontanelles, which has just been mentioned.

*Venous hyperœmia* of the brain occurs quite often in children, as in croup and laryngismus stridulus; also in diseases of the lungs, whether hereditary, as atelectesis, or acquired, in hereditary and acquired defects in the heart, etc.

In all these and similar cases venous hyperæmia may cause anæmia in the cortex of the brain. This may also occur during disturbed digestion, constipation with considerable generation of gas in the intestines; this forces the diaphragm upwards, and impedes the circulation of blood to and from the brain. I also wish to mention that a protracted fluxion of the brain produces a venous congestion in it, because the capillary vessels become compressed from the condensed cerebro-spinal fluid and the *vis a tergo* in the veins is diminished, and the blood flows more slowly.

The second group of symptomatic convulsions are those that are caused by a *change in the blood.* This is often combined with disturbances in the circulation, hence this group may have several co-operating causes. Among the changes in the blood that cause or promote convulsions are: 1st. *An abnormally increased temperature.* That this plays an important role among the causes of convulsion, especially during childhood, is scarcely questionable. The explanation of this fact is that the excitability in the nerve element increases by an increased temperature.

In a sudden case of fever in a child, especially an infant, it is often announced by a convulsion. This corresponds to the chill in the adult. Especially is this the case in the croupous inflammations of the lungs and pleura. It is not only the increased temperature that in a suddenly developed fever produces convulsions, but increased body heat. There is as a rule an increased action of the heart; this produces a fluxion to the brain, and this again, as we have just seen, many result in a convulsion. In the croupous pneumonia it is probably the impeded respiration that produces venous hyperæmia, and this in proportion contributes to a convulsion. Just so in pleurisy; but here there is intense pain which no doubt contributes to convulsions, and this may be partially explained by reflex action. The same is the case, for example, in otitis accompanied with convulsions, where certainly the pain is of great importance. We see, consequently, that in similar cases many causes co-operate to produce convulsions. Such is the case with another kind of blood change, viz.:

2*nd. Infectious Diseases.* Scarlatina, morbilli, variola, cerebro-spinal meningitis, intermittent fever, etc., are not infrequently accompanied with convulsions during the early stages. In those diseases we must assume that it is the qualitative blood change that reacts on the nervous system. But even if this is the chief cause *i. e.*, the changed condition of the blood, producing disturbed nutrition in the nervous system, we must still recognize other causes, viz.: increased temperature, which appears early, also disturbed circulation within the brain.

Consequently we see that many causes may co-operate to produce convulsions during the commencement of fevers. These convulsions occurring during later stages are most frequently caused by an anæmia of the brain from some cause or another, as just mentioned.

3d. Direct intoxication, through mineral and vegetable poisons; to these belong lead poisoning, atropia poisoning, tobacco enema, poisonous sponges, alcohol,* etc. To this class belongs poisoning through the inhalation of gases such as carbonic acid gas.

4th. Such changes in the blood, where a poison is taken up from the system, as in pyæmia, septicæmia, puerperal infection, etc. In these cases, as in direct poisoning, we must accept that the poison acts as an irritant to the nervous system, perhaps directly on the convulsive center. Here belongs carbonic acid gas poisoning, caused by a disturbed respiration, and the blood thus becomes overcharged with carbonic acid; this irritates the nervous system, and a convulsion results. In uræmic convulsions, whether they are caused by an increased amount of excrementitious material irritating the nervous system, or if caused by an acutebrain disease producing an anæmia of the cortex of the brain is not yet decided.

Here we may also mention changes in the milk of nurses and mothers; we may, indeed, include intoxications produced by sudden effects through the mind, viz: fear, wrath, etc., which in the child may produce convulsions. In what the change or intoxication of the milk depends is not known, but many cases of convulsions in children, originating from such causes are on record. Petit Rordel mentions a case where the mother, shortly after being suddenly angry, put her child to her breast with the consequence of convulsions. Bouchut mentions a similar case. Underwood has reported a case where a man was to make a visit to a family; just as he came inside the door he fell to the floor and died. The lady became very frightened, soon after put her six months old child to her breast, and within one hour the child had convulsions, alternating with coma, lasting thirty-six hours, then recovered. Saltman mentions a case somewhat similar.

The *Sympathetic* convulsions are those that are produced by

* Solman mentions a case where an infant raised by a wet nurse had frequent attacks for eight days without finding a cause. Finally a bottle of brandy was found in her bed. She was at once discharged and a new one accepted, and the child had no more convulsions.

reflex action. They may be produced by any irritation of the sensitive nerves of the skin or mucous membrane. They are very common during childhood, and probably induced by an increased irritability of the sensitive nerves, partially through a diminished or less developed power of the reflex inhibitory center of the brain.

Here belong, for instance, convulsions occurring from burns of the skin, large superficial ulcers, extensive intertrigo, etc., pointed foreign bodies that irritate the nerves of the skin as needles. Siebenhaar mentions a case of a child, nine years old, suffering from convulsions who was ordered a bath, when on taking off the clothing, a needle was discovered penetrating into the small of the back, deep in the flesh. The needle was removed, and convulsions ceased.

Irritations of the membrane of the ear, whether inflammatory or a foreign body in the auditory canal, may produce convulsions; also foreign bodies in the nasal passages, and, furthermore, an irritation of the kidneys or ureter, through renal calculi, not uncommon in children. Demme mentions a case of a boy, one year old, with hereditary constrictions of the urethra, causing great obstruction in urinating, which often produced convulsions. The urethra was dilated, and the convulsions disappeared.

The largest proportion of convulsions, however, are produced through an irritation of the mucous membrane of the stomach and intestines. That this cause produces convulsions is one of the oldest-known and acknowledged facts. We see them produced by dyspepsia, colics, intestinal catarrhs, gastro-enteritis, constipation, etc. In practice, we find many cases where the cause is hard to explain but after a thorough search we can generally find some ailment in the intestines; most common is chronic catarrh. This treated and improved, the convulsions will disappear. Especially has this been the case in rachitic children in my practice.

One question much disputed, and yet an open one, is, can couvulsions be produced by irritation from worms? Most authors regard it as perfectly natural that such a case exists. At one time it was regarded as the cause in most cases of convulsions; other authors, though few, deny any relation between the presence of worms and convulsions. To judge between them is difficult, for on the one side we see cases happen where children had worms, which through anthelmintics had been destroyed, and no convulsions followed. I have seen a child who discharged 100, others 50, 60 and 70 worms, without ever having had convulsions. On the other hand, cases are reported where we must accept, that convulsions are produced by their presence in the intestinal canal, and consequent irritation of the sensitive nerves.

Cormak mentions a case in a boy, seven and a half years of age, otherwise perfectly healthy, suddenly attacked with convulsions, which were cured after administering calomel and santonin, which discharged many ascarides. I have not seen a similar case. I therefore believe that we can not altogether disregard worms as one of the causes of convulsions, but we must also be careful in a case of convulsions, where worms are inhabiting the canal, to not too suddenly come to a conclusion, but search after other causes, which otherwise may be passed by.

Another question that also takes us back to antiquity, and on which modern thought yet can not agree is, can convulsions be produced by dentition or not? Among the old authors the majority acknowledge it to be a common cause, but we find many claiming convulsions to be independent of dentition, and thus, we may say, stands the question to-day. Most claim that convulsions may be produced by difficult dentition; others deny it. Dentition is said to be a phyisological process, and as such could or should not cause a dangerous complaint. Let it be so. Is there not any other physiological process that is accompanied with danger to an individual? Our very first entrance into this world gives us at once an answer to this question. Dentition is, however, a process, that, in some children, without doubt, is combined with pain, and this at a period when reflex action is easily produced; and that partially through the last branches of the dental nerves, and partially through the end nerves in the swollen gums, convulsions by reflection may be produced, appears to me not at all impossible. Physiology, indeed, tells us that reflex actions are easier produced from the end nerves than from the trunk; and, when we see cases, just before teething, otherwise healthy, with repeated attacks coming on, ceasing as protruded, only possible to begin again while cutting another tooth, and impossible to find any other cause, then I, at least, can not neglect to place dentition and convulsions with each other as cause and effect. I remember a case, now many years since, and then I was convinced, that dentition could not cause convulsions. I was called in consultation by one of my colleagues to see a child who had had several attacks. The parents, especially the father, were very calm, saying that the convulsions now, just as once before, probably depended on dentition. I did not believe it, but made as thorough search as I could for the cause, but in vain. After one or two days, during which time the convulsions continued, a tooth protruded, and the convulsions ceased. Other cases could be cited. What otherwise concerns dentition, we should remember that hereditary disposition plays also an important part. What, then, will not produce convulsions in one child, may very easily in another. It is very difficult, however, to certainly decide a case of convulsions as to cause, as many causes may be found. The one nearest at hand is irritation of the mucous membrane of the intestine, as it is just at this time that dentition takes place, when intestinal catarrhs are so common, and that this alone is capable of producing convulsions, we have just shown.

I mentioned disposition and hereditary tendency to convulsions. This topic also deserves to be touched upon in connection with causes to convulsions. Without doubt there is in certain families a disposition to convulsions of a hereditary nature. On what this disposition, this "convulsability," depends, we know not; whether from a disturbed nutrition within the nervous system, or some anomaly in the blood that affects the vaso-motor system; but it is, nevertheless, a fact supported by many reported cases. Duclos reports that a married lady, in her childhood, had suffered from convulsions up to her seventh year. They then ceased, but there remained a deviation in her mouth, and the eyelid of the left eye was prolapsed. She had had had nine brothers and sisters, of whom six died in convulsions. She was herself mother to ten children; all have had convulsions; six died; of these, five were under two years of age, and the sixth at three years.

Her first-born got convulsions after nursing when the mother shortly before had been angry.

Lastly, in childhood, and, of course, in older patients, cases of convulsions may occur which are simulated.—*Hygiea-Medecinsk och Farmacevtsk Mauadskrift.*

---

### EXPERIMENTAL STUDIES ON INTOXICATION WITH MERCURY.

M. Prevost has published his experiments on the action of mercury on the several organs. To avoid the irritation of the gastro-intestinal tube by mercury introduced into the stomach, the hypodermic injections have been recommended. But Prevost proves that these injections, wherever they are made under the skin, will provoke the same lesions in the intestines. These lesions are especially found in the cœcum and the large intestines, the mucous membrane of which is found to be in a state of hyperæmia, and covered by ecchymotic plaques. Walter Hay has recently proved that hypodermic injections of mercury cause irritation at the point of injection, with reflex irritation on the secretory and vaso-motor activity of the intestinal canal, if the injection is made in a part of the skin which is in nervous relation with the abdominal contents. Mercurial albuminuria has been thoroughly studied, and researches have shown a calcification of the tubuli in the kidneys. Experiments have been made on several animals, and recently, in a case of poisoning by acid nitrate of mercury, the same phenomena have been found. In certain cases of hydrargyrism, death is caused by total suppression of the renal functions. Osseous alterations have also been found to be due to application of mercury, and M. Prevost found decalcification of the bones which provoked, in some cases, mobility of the epiphyses of the long bones on their diaphyses. To prove that decalcification of the long bones exists, and goes hand-in-hand with the calcification of the tubuli in the kidneys, he extirpates one tibia of the animal before giving the mercury.—*L'Union Médicale.*

